# Micro-Hotspots of Risk in Urban Cholera Epidemics

**DOI:** 10.1093/infdis/jiy283

**Published:** 2018-05-11

**Authors:** Andrew S Azman, Francisco J Luquero, Henrik Salje, Nathan Naibei Mbaïbardoum, Ngandwe Adalbert, Mohammad Ali, Enrico Bertuzzo, Flavio Finger, Brahima Toure, Louis Albert Massing, Romain Ramazani, Bansaga Saga, Maya Allan, David Olson, Jerome Leglise, Klaudia Porten, Justin Lessler

**Affiliations:** 1 Departments of Epidemiology, Johns Hopkins Bloomberg School of Public Health, Baltimore, Maryland; 2 Departments of International Health, Johns Hopkins Bloomberg School of Public Health, Baltimore, Maryland; 3 Epicentre, Paris, France; 4 Institut Pasteur, Paris, France; 5 Communauté des Amis de l’Informatique pour le Développement, N’Djamena, Chad; 6 Ministry of Health, Kalemie, Democratic Republic of the Congo; 7 Department of Environmental Science, Informatics and Statistics, Università Ca’ Foscari Venezia, Venice, Italy; 8 Centre for the Mathematical Modelling of Infectious Diseases, London School of Hygiene & Tropical Medicine, United Kingom; 9 Médecins sans Frontières (France), Kinshasa, The Democratic Republic of the Congo; 10 Médecins sans Frontières (France), Kalemie, The Democratic Republic of the Congo; 11 Solidarités International, Paris, France; 12 Médecins sans Frontières, Paris, France

**Keywords:** cholera, clustering, ring vaccination, targeted interventions, vibrio cholerae

## Abstract

Targeted interventions have been delivered to neighbors of cholera cases in major epidemic responses globally despite limited evidence for the impact of such targeting. Using data from urban epidemics in Chad and Democratic Republic of the Congo, we estimate the extent of spatiotemporal zones of increased cholera risk around cases. In both cities, we found zones of increased risk of at least 200 meters during the 5 days immediately after case presentation to a clinic. Risk was highest for those living closest to cases and diminished in time and space similarly across settings. These results provide a rational basis for rapidly delivering targeting interventions.

Cholera epidemics in sub-Saharan Africa produce a large proportion of global cholera mortality and continue to wreak havoc on already fragile nations [1, 2]. Targeting cholera interventions to transmission hotspots, or areas of elevated transmission intensity in urban areas, may be the best control strategy when resources are constrained [3, 4]. In recent years, rapid response teams have been proposed as an important way to fight cholera in cholera-prone countries. These teams can quickly provide emergency water, sanitation, and hygiene interventions (eg, point-of-use water treatment and basic hygiene educational materials) and sometimes oral cholera vaccine to neighbors of cholera cases [5, 6]. However, there is limited evidence regarding the impact or optimal spatial scale of these targeted interventions.

Cholera transmission is thought to occur through 2 modes of exposure [1]: environmentally mediated exposure, often due to fecal contamination in the broader environment, and [2] “direct” exposure to an infected individual (eg, being served food directly contaminated by a case) [7]. The mix of environmentally mediated and direct exposure shapes the spatiotemporal distribution of cases within an epidemic. Evidence from Bangladesh and other locations have shown that direct transmission plays an important role in cholera transmission, leading to elevated risk when residing close to an incident case [8]. Likewise, those living close together will often share risk factors and access shared water sources [9].

Characterizing the small-scale spatiotemporal distribution of cholera cases in epidemics can provide new and useful insight into the mechanisms of transmission, ultimately highlighting a path for efficient targeted cholera control. In this study, we use high-resolution data from epidemics in 2 African cities separated by thousands of kilometers, N’djamena in Chad and Kalemie in Democratic Republic of the Congo (D.R. Congo), to estimate spatiotemporal windows of increased cholera risk among neighbors of incident cholera cases.

## METHODS

### Setting

Chad has experienced cholera outbreaks at least once every 4 years since the 1990s. Médecins Sans Frontières (MSF) assisted the Chad Ministry of Health (MoH) to respond to a cholera outbreak that started in mid-April 2011 in which most cases occurred in the capital, N’djamena, home to approximately 1 million people. On June 22, 2011, the MSF team, with the assistance of other collaborating agencies, began systematically collecting household coordinates through a home visit to each suspected cholera case presenting at one of the official cholera treatment centers/units in N’Djamena (Supplementary Figures S1 and S2). As the number of cases per day began to rapidly increase in early October, MSF modified their protocol to collect household coordinates for 1 of every 3 cases.

Kalemie is located on Lake Tanganyika in eastern D.R. Congo and serves as a large urban trading center for the region. Cholera tends to occur annually in Kalemie with a seasonal peak within the last few months of the year. In Kalemie, MSF has worked with the MoH on comprehensive cholera prevention and control strategies since 2008. From January 1, 2013 to January 15, 2014, MSF and the MoH collected the household coordinates for each suspected cholera case seeking care at the main diarrhea treatment center in Kalemie, Centre de Traitement de Maladie Diarrhéique (Supplementary Figures S1 and S3).

In both settings, suspected cases were defined using a modified World Health Organization case definition (acute watery diarrhea regardless of age). Teams in both countries were trained in the use of the global positioning system devices to ensure stable and accurate readings.

### Statistical Approach

To characterize the spatiotemporal clustering of cases, we calculated τ, a global clustering statistic estimating the relative risk of the next case occurring at a distance *d*, within *t* days after a suspected (“primary”) case presents at a health facility compared with the risk of the next case occurring anywhere in the population (ie, the entire city) during the same period. We calculated τ using the IDSpatialStats package [10], with a 50-meter moving window estimated every 10-meters (except for distances <50 meters; Supplementary Data). This statistic is robust to heterogeneities in how the population is distributed (ie, differences in population density across a city), and it has been shown to be insensitive to dependencies in reporting rates that may vary in space (eg, distance from health center) and time (eg, epidemic phase) [10, 11]. We calculated 95% confidence intervals (CIs) as the 2.5th and 97.5th quantiles from 1000 bootstrap replicates.

We focused on estimating τ at distances up to 500 meters from a primary case and within 5-day windows up to 30-days after a primary case presented to a facility. We considered the zones of increased risk around incident cases to extend until the 95% CIs of τ cross unity for at least 2 consecutive points (ie, ≥20 consecutive meters). Because this classification may underestimate the extent of the zones of increased risk due to small sample size, we calculated the median distance at which τ dropped below 1.2 (eg, minimum 20% elevated risk) for each bootstrap as an alternative measure. Because it is unlikely that a targeted public health response can be mounted the same day a case seeks care, we also estimated zones of increased risk around incident cases, excluding the day of case presentation (day 0). Code and data from these analyses are at https://osf.io/4fsnc/.

## RESULTS

In Kalemie, D.R. Congo, household coordinates were successfully recorded for 1077 of 1146 suspected cholera cases reporting to the main diarrhea treatment center from January 2013 to January 2014. In N’djamena, household coordinates were recorded for 1692 of the 4359 suspected cases reporting to healthcare facilities within the city. All case households were visited from June 22, 2011, and 1 in 3 randomly selected case households were visited from August to the end of the outbreak (December) due to logistical constraints.

### The First Five Days

Within the first 5 days after a suspected cholera case presented for care, the zone of increased cholera risk extended to at least 220 meters from the home of the suspected cases in Kalemie and 330 meters in N’Djamena. Zones of increased risk defined using an alternative definition (τ ≥ 1.2; Supplementary Table S1) were similar. Those living within 40 meters of another case (including those in the same household) had a 121.1-fold (Kalemie; 95% CI, 89.7–164.8) higher risk than the general population of becoming a cholera case within 5 days of the primary case in Kalemie and a 32.4-fold (95% CI, 25.3–41.0) higher risk in N’Djamena (Figure 2A and B). Those living 75–125 meters from a case had 2.0 (Kalemie; 95% CI, 1.0–3.2) and 3.9 (N’Djamena; 95% CI, 2.7–5.4) times the cholera risk in the 5 days after the initial case compared with those anywhere in the city.

Within these first 5 days after a case presented for care, the most elevated risk occurred up to 1 day after case presentation. The zone of elevated risk within 1 day of a primary case extended to at least 340 meters in N’Djamena (like the estimate for the first 5 days) but only 80 meters in Kalemie. During this time frame, those within 40 meters of a primary case had a 189.7-fold (Kalemie; 95% CI, 139.7–261.9) and 55.4-fold (N’Djamena; 95% CI, 42.3–72.4) increased risk of presenting as a cholera case compared with those anywhere in the cities. From 75 to 125 meters from a primary case, this risk decreased to 1.9 (95% CI, 0.7–3.6) in Kalemie and 5.9 (95% CI, 3.8–8.7) in N’Djamena.

**Figure 1.**
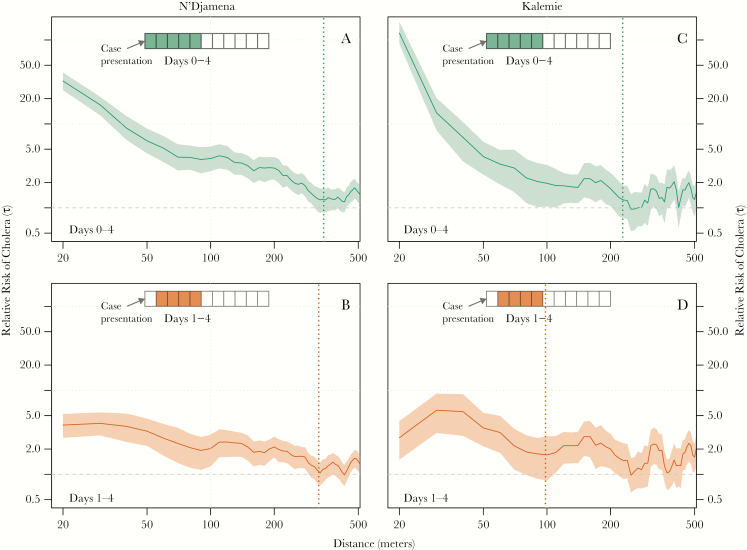
Estimates of the relative risk of the next cholera case being within a specific distance to another case (x-axis) within either days 0–4 (green, A and B) or days 1–4 (orange, C and D) compared with the risk of the case occurring anywhere in the population. Dashed lines represent the spatial extent of the zones of increased risk as defined by the first point at which the 95% confidence intervals cross unity over a 20-meter interval (ie, over 2 consecutive 10-meter points).

After excluding cases occuring the same day as a primary case (day 0), the zone of increased risk was 310 meters in N’Djamena (compared to 330 m for estimates including day 0) and 90 meters in Kalemie (compared to 220 m) (Figure 1C and D). During this time period, those within 40 meters of a primary case had a 2.7-fold (Kalemie; 95% CI, 1.5–4.3) and 3.9-fold (N’Djamena; 95% CI, 2.7–5.2) increased risk of presenting as a case compared with those anywhere in the cities. From 75 to 125 meters from a primary case, the risk was 1.7 (95% CI, 0.8–2.9) in Kalemie and 2.0 (95% CI, 1.2–3.0) in N’Djamena.

### Diminishing Risk Over Time

**Figure 2.**
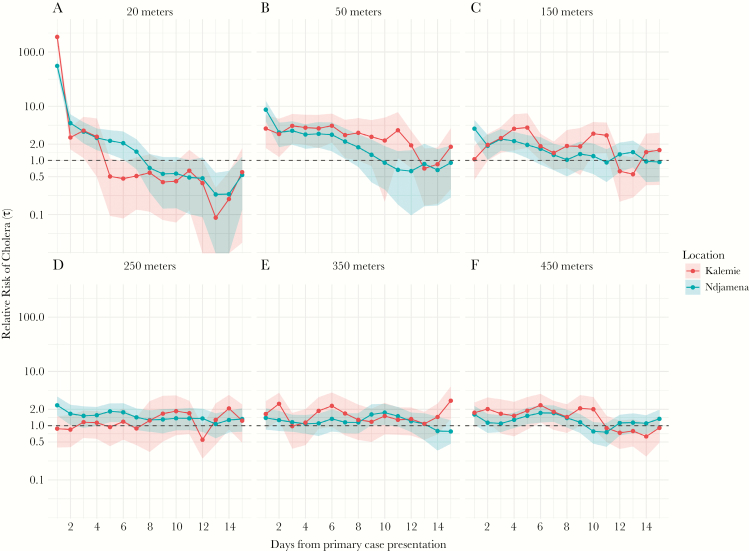
Estimates of the relative risk of the next cholera case occurring at different distances from a primary case (panels A–F illustrate 20–450 meters) compared with the risk of the case occurring anywhere in the population by time from primary case presentation (y-axis). N’Djamena estimates and 95% confidence intervals are shown in green and those from Kalemie are shown in orange.

In secondary analyses, we explored how the elevated risk changed with time at key distances away from primary cases’ households to better illustrate the dynamic increased risk zone. We find that at 20 meters, a scale likely representative of a household and/or first-degree neighbors, significant elevated risk disappeared by 3 (Kalemie) and 6 (N’Djamena) days after the presentation of the primary case (Figure 2A), with the relative risk point estimates remaining below 1 in both locations after 7 days. At 50 meters from the primary case household, risk remains elevated for slightly longer (~7 days). At 150 meters from the primary case household, the elevated risk period does not start until 2–3 days after case presentation, although it still ends by day 5–6 (Figure 2).

## Discussion

These results reveal similar spatiotemporal patterns of cholera cases across epidemics in 2 African cities. We find clear evidence for zones of increased risk extending at least 200 meters from the household of a cholera case within the first 5 days after he/she presents for care, with most elevated risk within the first days after the case and within 100 meters of the household. Although not as dramatic, these zones of risk persist even after excluding those cases that appear on the same day. These suggest that interventions focused within at least 100 meters around cases’ households implemented within 1 week of case presentation may be an efficient cholera control strategy.

Although the similarity in the spatiotemporal structure between 2 independent settings provides reassurance that these results reflect some shared biological or structural properties of cholera transmission, there are several limitations to these analyses. First, we relied on suspected cases seeking care at health facilities. Analyses of epidemics in similar settings have shown that the true proportion of confirmed cholera cases among suspected cases can vary widely, and we expect that this misclassification would often dilute the risk ratios, biasing results towards the null, except in cases with a simultaneous outbreak of a directly transmitted disease. On the other hand, people living near suspected cases might be more likely to seek care due to concerns about cholera, which could create an upward bias. In N’Djamena, we only collected a random subset of cases household locations at the end of the epidemic; however, secondary estimates of clustering at different points in the epidemic suggest no major differences (Supplementary Figures S4 and S5). If cases within households (or neighbors) tended to seek care close together in space and time relative to the case sampling fraction in N’Djamena, we may have captured too few pairs of “related cases”, thus biasing our estimates of spatial risk towards unity. Although there were likely people who did not seek care with mild and asymptomatic cholera, if they were randomly distributed with respect to their spatiotemporal distance from another case, our estimates would vary little. However, if there was spatiotemporal clustering in nonreported cases, our results could be biased in either direction. Although τ is related to the more commonly used K-function and pair-correlation functions [11], it allows us to estimate second-order clustering without information on the underlying population structure but instead with data on the natural history of cholera (eg, the serial interval) and the overall distribution of cases. Uncertainty in the assumed serial interval was not captured in our estimates. Finally, given that we are dividing our data into spatial and temporal windows, the sample size can get small, and we may not have the power to detect low levels of elevated risk. Thus, the true zones of increased risk may be larger than suggested by τ's 95% CIs.

Case-area targeted interventions are not a new concept and have been implemented for diseases such as polio and smallpox. In many cholera epidemics, case-area targeted interventions, ranging from hygiene promotion to antibiotic prophylaxis, are part of the standard protocol, although they are rarely documented or evaluated in published literature [12, 13]. Careful evaluation of the timing, extent, and type of case-area targeted interventions are warranted. These interventions may not be ideal across all settings based on both the available human and other resources (eg, median 1011 people in 200-meter rings in N’Djamena; Supplementary Figure S5) and the epidemic dynamics. Identifying when (eg, lull periods between epidemics [14]) and where case-area targeted interventions may have the biggest impact is key.

## Conclusions

These results shed new light on the small-scale spatial structure of cholera transmission and point towards the possibility of conducting effective and efficient targeted interventions in urban cholera epidemics. Although the effectiveness of case-area targeted interventions will depend both on the types of interventions and the speed at which they are delivered, this work serves as rational for their use.

## Supplementary Data

Supplementary materials are available at *The Journal of Infectious Diseases* online. Consisting of data provided by the authors to benefit the reader, the posted materials are not copyedited and are the sole responsibility of the authors, so questions or comments should be addressed to the corresponding author.

Supplemental Figures and Tables

Supplementary Text

## Notes

***Financial support.*** The original field work was funded by Médecins sans Frontières as part of their operational programs. Support for the analyses described in this manuscript came from the Bill and Melinda Gates Foundation (OPP1171700, OPP1127318, OPP10892431,OPP1148763) and the National Institutes of Health (R01 AI102939; to J. L.). F. F. acknowledges support through the Swiss National Science Foundation Early Postdoc Mobility Fellowship (P2ELP3_175079).

***Potential conflicts of interest.*** All authors: No reported conflicts of interest. All authors have submitted the ICMJE Form for Disclosure of Potential Conflicts of Interest.

Presented in part: US Japan Cholera Meeting, 2015, Bethesda, MD.

## References

[1] Abubakar A, Azman AS, Rumunu J, et al The first use of the global oral cholera vaccine emergency stockpile: lessons from South Sudan. PLoS Med 2015; 12:e1001901. 26576044 10.1371/journal.pmed.1001901 PMC4648513

[2] Abubakar A, Almiron M, Charito A, et al Cholera, 2014. Wkly Epidemiol Rec 2015; 90:517–44.26433979

[3] Azman AS, Luquero FJ, Rodrigues A, et al Urban cholera transmission hotspots and their implications for reactive vaccination: evidence from Bissau city, Guinea bissau. Vinetz JM, editor. PLoS Negl Trop Dis 2012; 6:e1901. 23145204 10.1371/journal.pntd.0001901 PMC3493445

[4] Rebaudet S, Sudre B, Faucher B, Piarroux R Cholera in coastal Africa: a systematic review of its heterogeneous environmental determinants. J Infect Dis 2013; 208:S98–106. 24101653 10.1093/infdis/jit202

[5] Parker LA, Rumunu J, Jamet C, et al Neighborhood-targeted and case-triggered use of a single dose of oral cholera vaccine in an urban setting: feasibility and vaccine coverage. Kosek M, editor. PLoS Negl Trop Dis 2017; 11:e0005652. 28594891 10.1371/journal.pntd.0005652 PMC5478158

[6] Santa-Olalla P, Gayer M, Magloire R, et al Implementation of an alert and response system in Haiti during the early stage of the response to the cholera epidemic. Am J Trop Med Hyg 2013; 89:688–97. 24106196 10.4269/ajtmh.13-0267 PMC3795099

[7] Morris JG Jr Cholera–modern pandemic disease of ancient lineage. Emerg Infect Dis 2011; 17:2099–104. 22099113 10.3201/eid1711.111109 PMC3310593

[8] Sugimoto JD, Koepke AA, Kenah EE, et al Household transmission of *Vibrio cholerae* in Bangladesh. PLoS Negl Trop Dis 2014; 8:e3314. 25411971 10.1371/journal.pntd.0003314 PMC4238997

[9] Bi Q, Azman AS, Satter SM, et al Micro-scale spatial clustering of cholera risk factors in urban Bangladesh. PLoS Negl Trop Dis 2016; 10:e0004400. 26866926 10.1371/journal.pntd.0004400 PMC4750854

[10] Lessler J, Salje H, Grabowski MK, Cummings DA Measuring spatial dependence for infectious disease epidemiology. PLoS One 2016; 11:e0155249. 27196422 10.1371/journal.pone.0155249 PMC4873007

[11] Salje H, Lessler J, Endy TP, et al Revealing the microscale spatial signature of dengue transmission and immunity in an urban population. Proc Natl Acad Sci U S A 2012; 109:9535–8. 22645364 10.1073/pnas.1120621109 PMC3386131

[12] Guévart É, Noeske J, Sollé J, Mouangue A, Bikoti JM Antibioprophylaxie ciblée à large échelle au cours de l’épidémie de choléra de Douala en 2004. [Cahiers d’études et de recherches francophones/Santé] 2007; 17:63–8. 17962152

[13] George CM, Monira S, Sack DA, et al Randomized controlled trial of hospital-based hygiene and water treatment intervention (CHoBI7) to reduce cholera. Emerg Infect Dis 2016; 22:233–41. 26811968 10.3201/eid2202.151175 PMC4734520

[14] Rebaudet S, Gazin P, Barrais R, et al The dry season in Haiti: a window of opportunity to eliminate cholera. PLoS Curr 2013; 1. doi:10.1371/currents.outbreaks.2193a0ec44 01d9526203af12e5024ddc. 10.1371/currents.outbreaks.2193a0ec4401d9526203af12e5024ddc PMC3712488 23873011

[15] Finger F, Bertuzzo E, Luquero FJ, et al The potential impact of case-area targeted interventions in response to cholera outbreaks: a modeling study. PLoS Med 2018; 15:e1002509. 29485987 10.1371/journal.pmed.1002509 PMC5828347

