# Supplementary material for: Micro-Hotspots of Risk in Urban Cholera Epidemics
Source: J Infect Dis. 2018 May 11;218(7):1164–8. doi: 10.1093/infdis/jiy283 (PMC6107744; doi:10.1093/infdis/jiy283)
Supplement: Supplemental Figures and Tables [file jiy283_suppl_supplemental_figures_and_tables.docx]

**Supplemental Figures and Tables:**


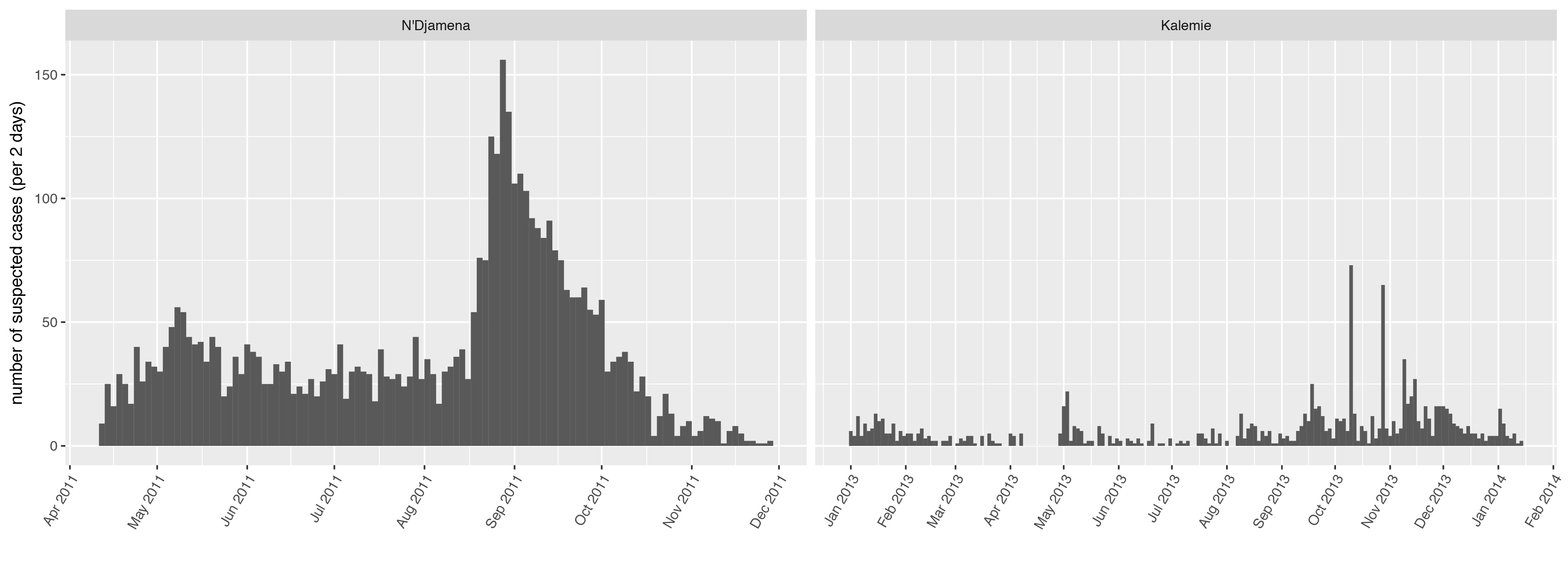


Figure S1. Epidemic curves from Ndjamena, Chad and Kalemie, the Democratic Republic of the Congo (A and B).


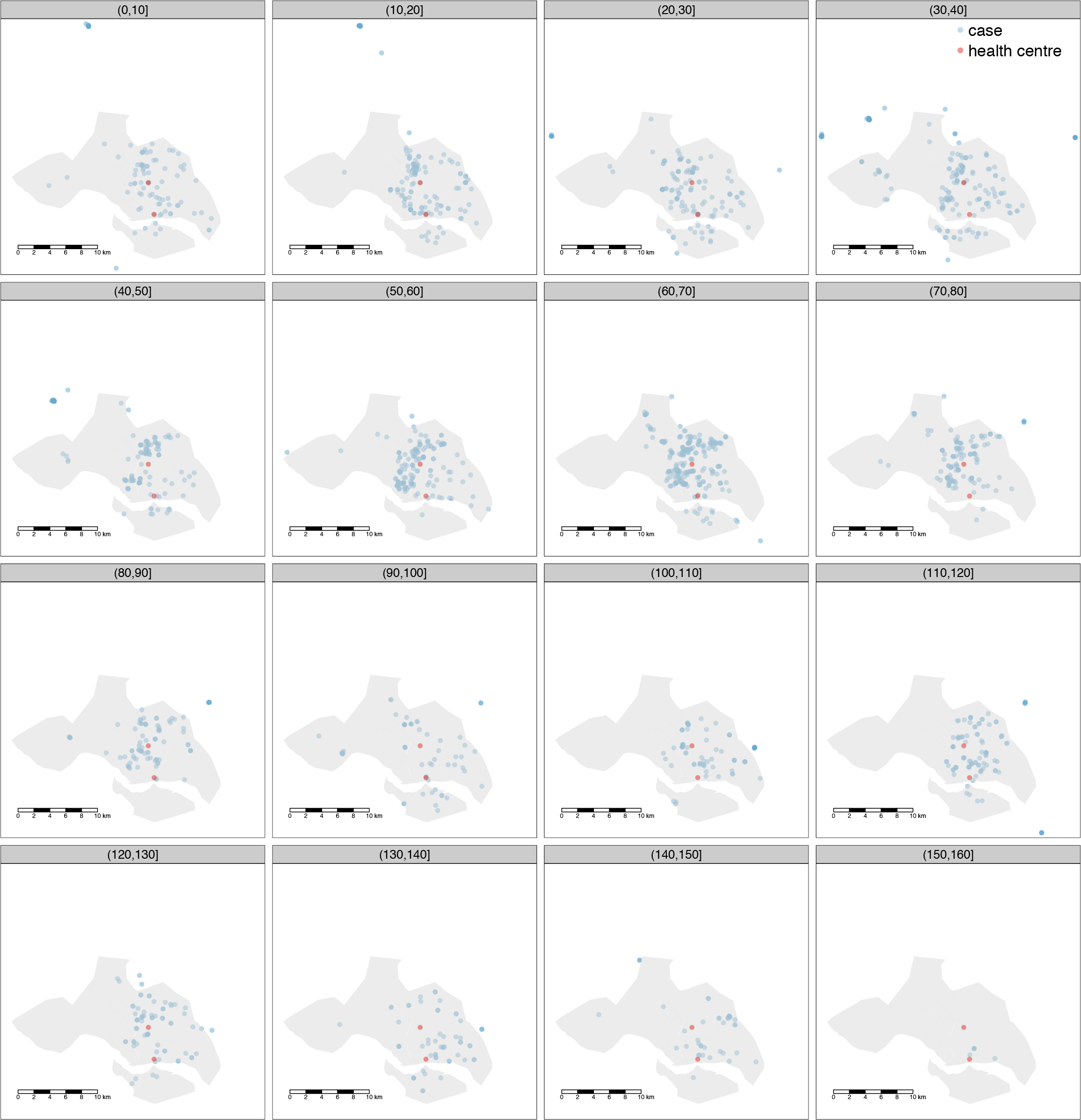


Figure S2. Map of cases by 10-day window in N’djamena, Chad. Blue dots represent cases and red dots represent the cholera treatment centres. Approximate boundaries of the city are shown in grey. Note that day-1 is June-22-2011.


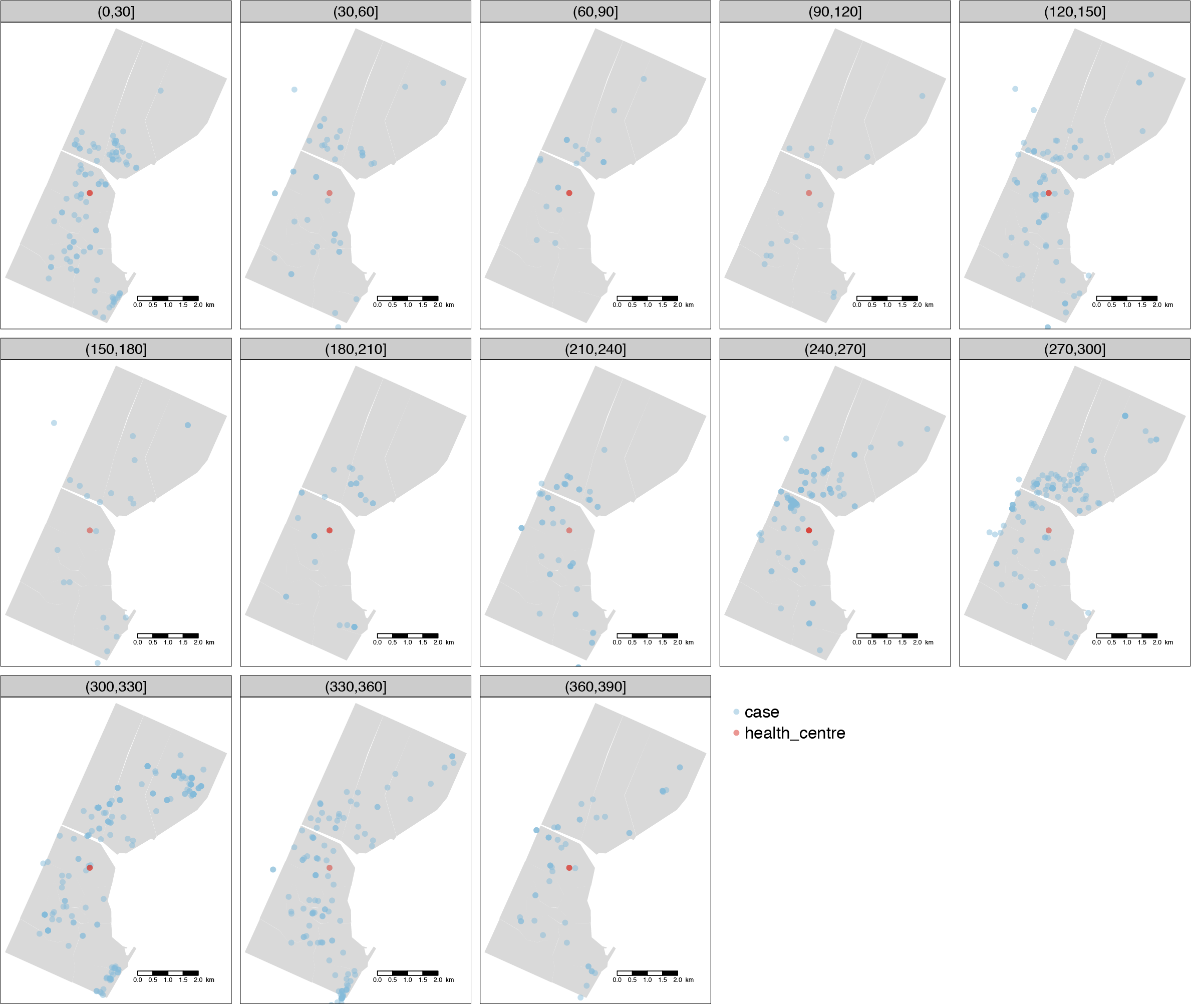


Figure S3. Map of cases by 30-day window in Kalemie, Democratic Republic of the Congo. Blue dots represent cases and red dots represent the cholera treatment centre. Approximate boundaries of the city are shown in grey.


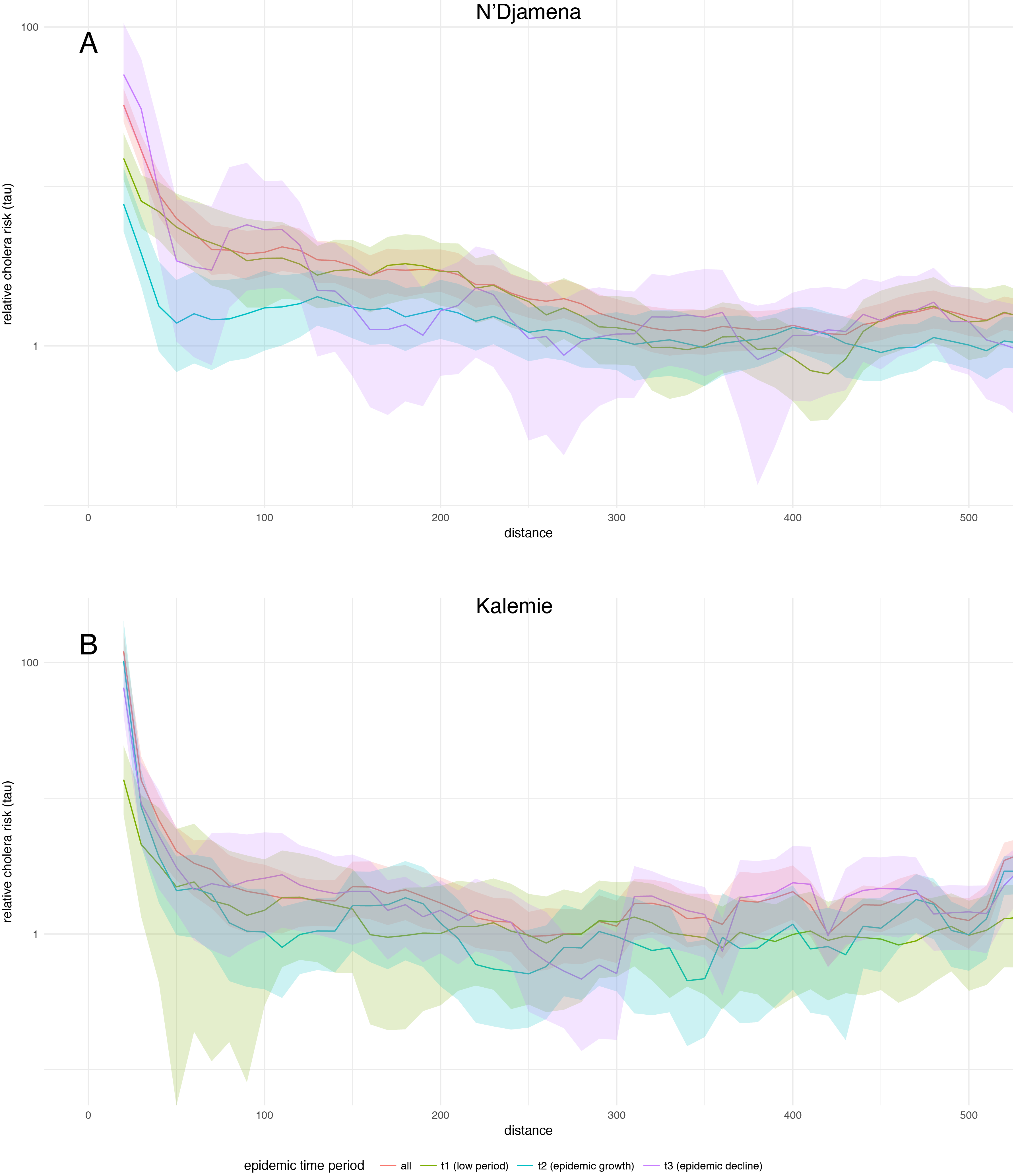


Figure S4. Estimates of τ and bootstrap 95% confidence intervals by epidemic phase in N’Djamena, Chad and Kalemie, the Democratic Republic of the Congo. The low period (t1) was from 1-Jan-2013 to 20-July-2013 for Kalemie and 22-Jun-2011 through 10-August-2011. The epidemic growth period (t2) was 21-July-2013 to 28-October-2013 for Kalemie and 11-August-2011 through 9-September-2011 in N’Djamena. The epidemic decline period (t3) started from 22-July-2013 in Kalemie and 10-September-2011 in N’Djamena through the end of each city’s outbreak.


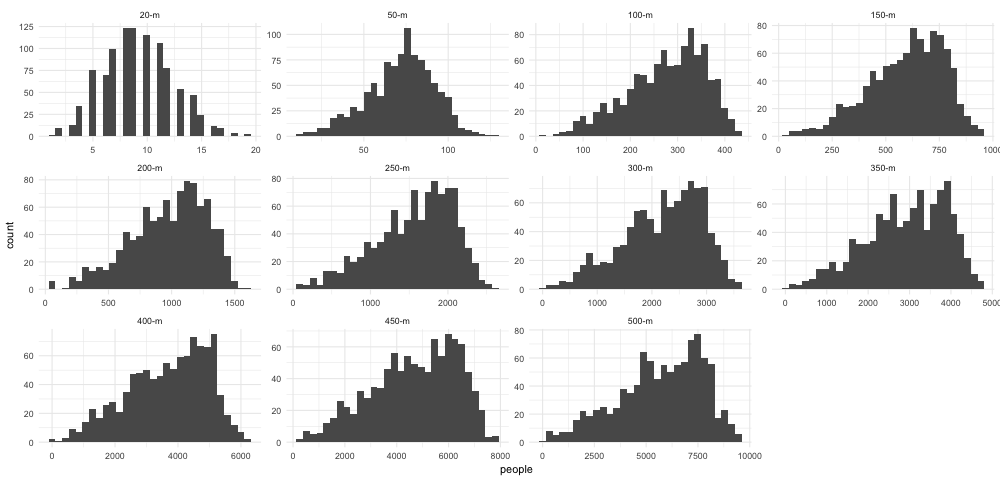


Figure S5. Median number of people per ring in N’djamena. Estimates based on remotely sensed built-up density where each of 993,500 inhabitants were randomly assigned to a 30m by 30m grid-cell with probability proportional to the estimated average built-up density of the cell (15).

Table S1. Median extent of the zone of increased risk based on the (bootstrap) distribution of maximum distances where $\tau$ was greater than 1.2. 95% confidence intervals represent the 2.5^th^ and 97.5^th^ percentiles of the bootstrap distribution.

|  | Median extents of zone of increased risk (days 0-5), 95% CI | Median extents of zone of increased risk (days 1-5), 95% CI |
| --- | --- | --- |
| Kalemie | 230m (90-420) | 250m (80-590) |
| N’Djamena | 365m (290-810) | 315m (155-430) |
