# Supplementary material for: Micro-Hotspots of Risk in Urban Cholera Epidemics
Source: J Infect Dis. 2018 May 11;218(7):1164–8. doi: 10.1093/infdis/jiy283 (PMC6107744; doi:10.1093/infdis/jiy283)
Supplement: Supplementary Text [file jiy283_suppl_supplementary_text.docx]

***Micro-hotspots of Risk in Urban Cholera Epidemics: Supplementary Text***

Andrew S. Azman, Francisco J. Luquero, Henrik Salje, Nathan Naibei Mbaïbardoum, Ngandwe Adalbert, Mohammad Ali, Enrico Bertuzzo, Flavio Finger, Brahima Toure, Louis Albert Massing, Romain Ramazani, Bansaga Saga, Maya Allan, David Olson, Jerome Leglise, Klaudia Porten, Justin Lessler

**The** [**
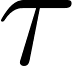
**](about:blank)**-function as a measure of spatial clustering:**

We used the [
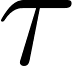
](about:blank) function as a measure of spatial dependence of cases within this study. This statistic, which can be interpreted as a relative risk, does not require information on the underlying spatial structure of the population and does not require edge corrections, like other commonly used measures of spatial dependence, like the related K-function [[1]](https://paperpile.com/c/GvTzOO/gbG5). Specifically, following the notation of Lessler et al [[2]](https://paperpile.com/c/GvTzOO/57fn), [
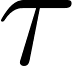
](about:blank) is defined as:

[
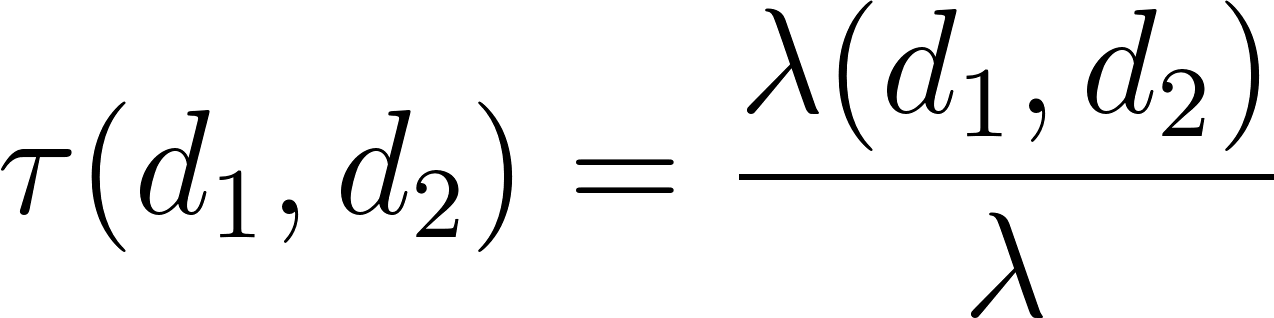
](about:blank),

where [
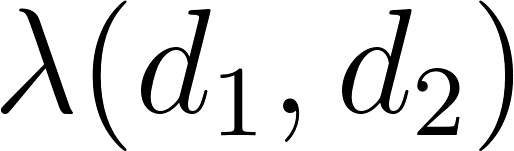
](about:blank) is the expected incidence rate of of people living between distances [
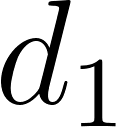
](about:blank) and [
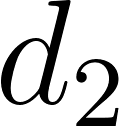
](about:blank) of a primary case and [
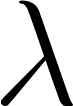
](about:blank) is the incidence rate in the entire population. When the underlying population structure is known, these incidence rates can be easily calculated. However, in many situations, like the present study, the structure of the population is not known. For these situations, we rely on an additional source of data, in this case the time between cases (i.e., the approximate serial interval of cholera) to help distinguish cases that are potentially closely related to one another. We use the following estimator for [
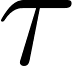
](about:blank):

[
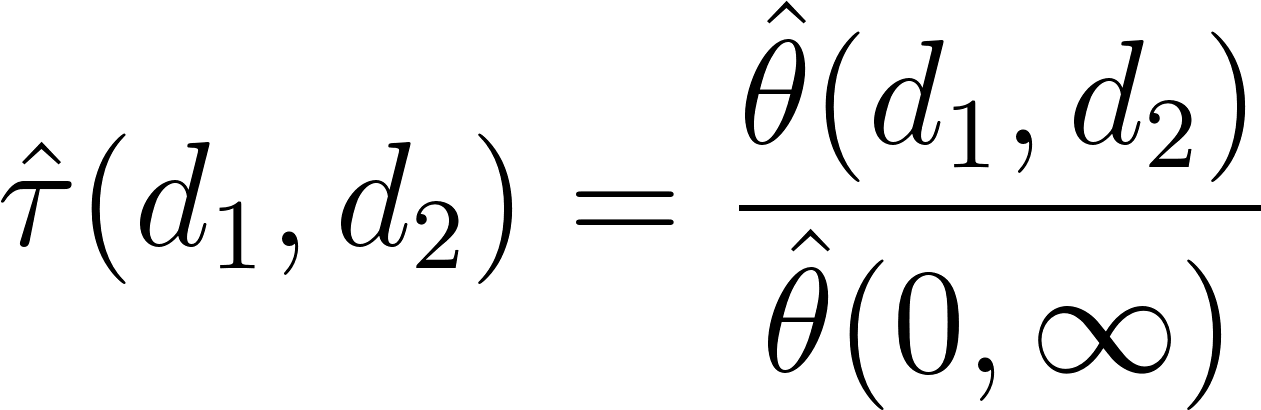
](about:blank),

Where [
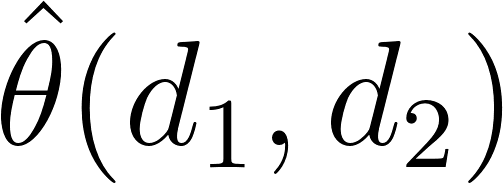
](about:blank) represents the odds that a potentially related case occurs within [
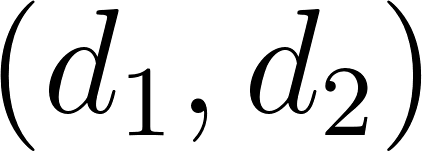
](about:blank) of another case. In our study, we considered cases that occured from 0 to 5 days after a primary case as potentially related. Thus our estimates of the
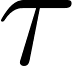
-statistic can be interpreted as the relative risk that a person living between [
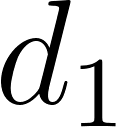
](about:blank) and [
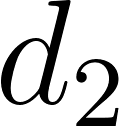
](about:blank) from an incident suspected cholera case (referred to as the primary case) becomes a reported suspected case between 0 and 5 days (approx. serial interval where we assume cases are potentially related) after the primary case compared to the risk of a case occurring anywhere in study area during that time period.

**Estimation of** [**
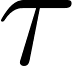
**](about:blank) **in this study:**

We used the IDSpatialStats R package (0.2.2) to estimate [
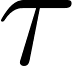
](about:blank). In the primary analyses we estimated [
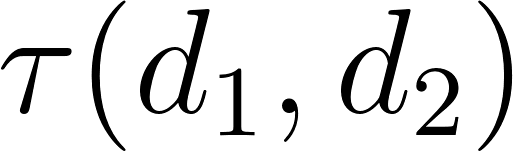
](about:blank) for [
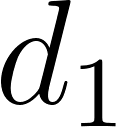
](about:blank) starting at 5m and [
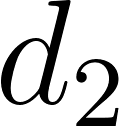
](about:blank) starting at 55m, incrementing the start and end of this 50m window by 10m until [
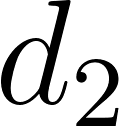
](about:blank) reached 525m. To get an estimate of clustering within a smaller spatial window, we also estimated [
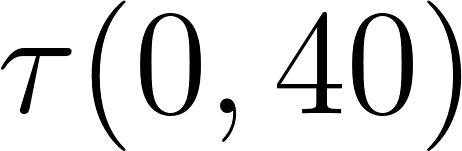
](about:blank). We estimated [**
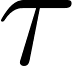
**](about:blank) for time windows of 0-4 and 1-4 days in the primary analyses in addition to a finer grid with a window size of 2 days, starting with a window from 0-2 days through 18-20 days.

This relatively small 50m moving window helps ensure that clustering at one distance will have minimal effect on clustering at other distances. However, this means that our estimates of [
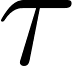
](about:blank) are not necessarily smooth or monotonic.

To estimate confidence intervals for [
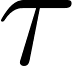
](about:blank) we used the bootstrap routine within the IDSpatialStats package, which excludes self-comparisons within the estimation of [
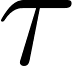
](about:blank) [[2]](https://paperpile.com/c/GvTzOO/57fn).

**References:**

1. [Waller LA, Gotway CA. Applied Spatial Statistics for Public Health Data. John Wiley & Sons; 2004.](http://paperpile.com/b/GvTzOO/gbG5)

2. [Lessler J, Salje H, Grabowski MK, Cummings DAT. Measuring Spatial Dependence for Infectious Disease Epidemiology. PLoS One. **2016**; 11(5):e0155249.](http://paperpile.com/b/GvTzOO/57fn)
